# Effect of Starter Culture and Low Concentrations of Sodium Nitrite on Fatty Acids, Color, and* Escherichia coli* Behavior during Salami Processing

**DOI:** 10.1155/2018/5934305

**Published:** 2018-10-04

**Authors:** Carla María Blanco-Lizarazo, Indira Sotelo-Díaz, José Luis Arjona-Roman, Adriana Llorente-Bousquets, René Miranda-Ruvalcaba

**Affiliations:** ^1^Agroindustrial Process Research Group, University of La Sabana, Colombia; ^2^Titular Professor, EICEA, Agroindustrial Process Research Group, University of La Sabana, Colombia; ^3^Engineering and Technology Department, Faculty of Advanced Studies Cuautitlán, National Autonomous University of Mexico (UNAM), Mexico; ^4^Chemistry Department, Faculty of Advanced Studies Cuautitlan, National Autonomous University of Mexico (UNAM), Mexico

## Abstract

The reduction of NaNO_2_ and safety in meat products have been a concern to the meat industry for the last years. This research evaluated the changes in total fatty acids (TFAs) and myoglobin forms by adding starter culture (*Lactobacillus sakei/Staphylococcus carnosus*) and 50 ppm of NaNO_2_ during salami processing. In the postripening stage, the starter culture influenced the concentration of the palmitic, oleic, vaccenic, and *γ*-linolenic TFAs, whereas the metmyoglobin concentration was lower (which could be related to the antioxidant effect of the starter culture). In this stage, an increase in enthalpy, specific heat, and onset temperature was found when adding starter culture and NaNO_2_, which is directly related to polyunsaturated TFA. However, when adding just the starter culture without 50 ppm NaNO_2_, the* E. coli *population was reduced in 4 log CFU/g. This study proposes the analysis of changes in meat product processing like salami in a holistic form, where the application of starter culture with low nitrite concentrations could be in the meat industry an upward trend for reducing this additive.

## 1. Introduction

During the processing of salami, physical, physicochemical, biochemical, and microbiological transformations occur. These changes are influenced by factors such as raw material, sodium nitrite, and starter culture. This process has an impact on the total fatty acid profile and it increases the unsaturation level and the susceptibility of oxidation [1, 2]. Furthermore, a concentration of 50 ppm sodium nitrite contributes in delaying lipid oxidation in meat products as a result of its reaction with iron [3]. The addition of NaNO_2_ influences the formation of the bright red color that corresponds to the nitrosylmyoglobin complex. Moreover, the addition of starter cultures influences changes in the concentration and types of fatty acids and oxidative phenomena of the lipid fraction [4].

Lipid oxidation, including autoxidation and enzyme-catalysed oxidation of fatty acids in fermented and dry-cured meat products, is involved in several aspects of meat products quality (functional, sensory, and nutritional) [5, 6]. Primary products of lipid oxidation generate changes in myoglobin such as the oxidation of oxymyoglobin (OMB) to metmyoglobin (MMB) inducing color changes [1, 7]; these changes result in a decrease in heme redox stability, rather than the oxidation of specific amino acid residues. This reaction generally proceeds in parallel to the lipidic oxidation, where the products of both reactions can mutually accelerate pigmentation and lipid oxidation [2, 8].

In addition, during the processing of salami a_w_ and pH are reduced; as a result, in these products foodborne pathogens are inhibited. However, deviations in the process parameters (temperature and/or humidity) affect the assurance of the elimination of food-borne pathogens in the final products [9, 10]. As a consequence, it is of great importance for the safety of the product the addition of starter cultures and the validation of the reduction of* E. coli *during salami processing.

Therefore, the design of the starter culture is critical for salami quality, where the addition of lactic acid bacteria (LAB) is aimed to ensure product safety and coagulase-negative staphylococci (CNS) is related with formation and stabilization of color and prevention of rancidity due to nitrate reductase and catalase activity [11]. In fact, the LAB* Lactobacillus sakei *and the CNS* Staphylococcus carnosus *have been selected as starter culture for their technological properties, where* L. sakei* decrease the pH through the fermentation of carbohydrates [12]. Furthermore,* L. sakei *produces NO and N_2_O due to its nitrite reductase and catalase activity heme independent [13]. In addition,* S. carnosus* can inhibit the oxidation of fatty acids due to its high nitrate reductase activity, which generates nitrites (NO_2_^−^) with antioxidant properties, taking part in nitrosomyoglobin formation, being involved in the development of color in fermented meat products [11].

Differential scanning calorimetry (DSC) is a thermo analytical technique that has been applied for lipid characterization. Thermal properties reported good relation with major triacylglycerols (TAG) and total fatty acids (TFA) and minor components free fatty acids (FFA), and oxidation products [14]. Multiple melting behaviors are explained by the melting of TAG and TFA with different melting peaks and crystal reorganization effects; also, enthalpy transitions are coupled with each other and strongly overlap. Consequently, the objective of this study was to evaluate changes of total fatty acids profile by CG-EM and MDSC, and myoglobin forms, during salami processing led by starter culture and the addition of 50 ppm of NaNO_2_. Furthermore, the reduction of* E. coli *was validated in salami with starter culture addition.

## 2. Materials and Methods

### 2.1. Salami Preparation

Four treatments were designed for the preparation of salamis (30 samples for each treatment): control (C) without the addition of the starter culture or NaNO_2_; control (C^+^) without the starter culture and 50 ppm of NaNO_2_; and treatments with the starter culture without NaNO_2_ (*Ls-Sc*) and with the starter culture and 50 ppm NaNO_2_ (*Ls-Sc*^+^).

Each treatment was evaluated in 3 different batches. Several salami samples units of 25±1 g each were prepared, the experimental samples were designed from mixtures based on pork meat (42% w/w) and pork back fat (23% w/w) under the formulation and procedures of a local industry (Bogotá, Colombia).

A commercial starter culture composed of* Lactobacillus sakei *and* Staphylococcus carnosus* (T-SC-150 Bactoferm™, CHR-Hansen, Hoersholm, Denmark) was added to the* Ls-Sc *and* Ls-Sc*^+^. The starter culture was previously activated in a 2% w/v dextrose (Scharlau Chemie SA, Barcelona, Spain) solution in sterile water and incubated at 25±0.1°C for 20 h. For every 10 kg of the mixture, 2.5g was added. The meat and fat were ground through a 10 mm diameter and mixed with 2.5% m/m NaCl, 0.1% m/m sodium erythorbate and NaNO_2_ according to the treatment and then stuffed into natural casings of 5 cm diameter.

Salami processing was performed at 80% of relative humidity in 4 stages: (i) the prefermentation stage, the first 12 h at 4±0.1°C; (ii) the postfermentation stage, the following 48 h at 21±0.5°C; (iii) ripening up to 14 days at 17±0.5°C. (iv) The last day of processing was considered post-ripening.

Measurements of pH and a_w_ were done according to protocols described by Blanco–Lizarazo et al. [15]. The initial pH for all treatments was 5.89±0.14 and in postripening stage was for* Ls-Sc *4.67±0.01,* Ls-Sc*^+^ 4.85±0.02, and C 4.43±0.01 and for C^+^ 4.52±0.04. The initial a_w_ was 0.910±0.12 and in postripening stage was 0.810±0.03 for all treatments.

### 2.2. Determination of Total Fatty Acids

Fatty acids were determined on the lipid extract from the salami samples at different processing times. Samples of 10g of salami were placed within an extraction cartridge and inserted into a reflux flask using petroleum ether under the Soxhlet methodology during 5 h. The obtained extracts were concentrated using a vacuum evaporator at 45°C.

Extracted lipids were methylated and transesterified using sodium methoxide at pH 3, according to the method of Park and Goins [16]. Subsequently, twice the volume of a saturated sodium chloride solution and 3 times the volume of ethyl acetate was added in a separatory funnel. Finally, 5g of anhydrous sodium sulfide was added and decanted. The samples were distilled at 40°C in vacuum.

The fatty acids methyl esters obtained from the TFAs and triglycerides of fatty acids were separated and quantified with a gas chromatographer (Agilent model 62630A, China) coupled to a mass spectrometer model 5975C with the Agilent triple axis detector (USA). The GC-MS system was equipped with a DB-5 column (Agilent, USA, 30 m x 0.25 mm x 0.25 *μ*m). Helium was used as the carrier gas at a flow rate of 1.0 ml/min, and the injection ratio was 1:10. The oven temperature was adjusted to 150°C with a holding time of 2 min, an initial ramp of 5°C/min up to 200°C, followed by a ramp of 3°C/min up to 215°C, and finally a ramp of 10°C/min up to 260°C, which was held for 2 min. The injector and detector temperatures were adjusted to 250°C and 290°C, respectively, and the injection volume was 1 *μ*l. The fatty acids methyl esters were identified by comparison of the respective retention times and analysis of their corresponding mass spectra with a mass spectral database of fatty acid methyl esters, ISBN: 978-1-1181-4394-0, MSD ChemStation (E.02.00.493, Agilent, USA) Software, (November 2011). Quantification was performed through areas under the curve of each peak based on total methyl esters. All analyses were performed by triplicate.

### 2.3. Modulated Differential Scanning Calorimetry (MDSC) Analysis of Salami Lipids

For the analysis of nonisothermal transitions, 5±0.01 g samples of salami of the treatment* Ls-Sc*^+^ were collected at 0 and 14 days. To ensure the homogeneity and reproducibility of the sample, the 10 samples for each treatment were subjected to cryomilling with liquid nitrogen for 1 min and were milled by impact for five cycles of 10 s each with a grinder (IKA A11 Basic S1, USA).

A Modulated DSC 2920 (TA Instruments, New Castle, DE, USA) was used. A total of 12±1 mg of sample was placed in a hermetically sealed aluminum cell. The experimental conditions for the samples are based on the back fat pork analysis proposed by Sasaki et al. [17], with an initial temperature of 10°C and a final temperature of 100°C, a rate (ß) of 5°C.min-1, and modulation of ±0.796°C min-1. An empty cell was used as the reference. Indium was used for the temperature calibration, and Sapphire was used for the heat capacity.

### 2.4. Measurement of the Reflectance for Determinate Concentrations of Myoglobin Forms

Nine 17 mm thick samples were evaluated with 5 replicates for each salami processing time (0, 1, 2, 3, 7, and 14 days). The reflectance was measured between 400 nm and 700 nm in 10 nm intervals in accordance with protocols proposed by the American Meat Science Association [18] with the ColorquestXE spectro-colorimeter (HunterLab, Reston, USA) (light source A, standard geometric observation at 10° to 1 in of distance, and white standard). The concentrations of the chemical forms of myoglobin were calculated according to the protocols proposed by Tang* et al.* [19]. The reflectance values (503, 557, and 582 nm) were calculated using linear interpolation.

### 2.5. Microbial Analysis during the Processing of Salami

#### 2.5.1. Bacterial Growth

For the analysis of bacterial growth, 25±0.1 g of the sample was taken at 0, 3, 6, 9, 12, 16, 20, 24, 48, 72, 240, and 336 h. Then, 250 ml of 0.1% peptone water was added (Merck, Darmstadt, Germany) and homogenised for 120 s using a Stomacher (BA7021, Serward, England). Six 10-fold serial dilutions were performed with 0.1% peptone water and deep cultured in PCA agar plates (Scharlau Chemie S.A., Barcelona, Spain). This step was performed in triplicate and incubated at 37±1°C for 48 h to obtain the total bacterial count. LAB were cultured in Man Rogosa and Sharpe agar (MRS) (Scharlau Chemie S.A., Barcelona, Spain) and CNS in Baird Parker with egg yolk and potassium tellurite (Scharlau Chemie S.A., Barcelona, Spain).

#### 2.5.2. *E. coli* Reduction

For the analysis of* E. coli* reduction, salami samples for the treatments* Ls-Sc *and* Ls-Sc*^+^ were inoculated with 2.5±0.1 ml (4.4±0.19 Log CFU/g) of* E. coli *ATCC 25922™ previously activated with glass beads in 100 mL of BHI medium (Scharlau Chemie SA, Barcelona, Spain) and incubated (Friocell 22 - Comfort, MMM group, Munich, Germany) for 12 h at 37±1°C. The strain was maintained in glass beads with glycerol/Nutrient Broth (Scharlau Chemie SA, Barcelona, Spain) (20/80%) (v/v) and stored at -80°C in liquid nitrogen. The salami samples inoculated with* E. coli *were cultured in Eosin methylene blue agar (EMB) under identical conditions described above for analysis of bacterial growth.

#### 2.5.3. Modelling of Bacterial Behavior

To analyse the bacterial growth during the processing of salami the model developed by Baranyi and Roberts was applied [20]. This model was selected to determine the maximum growth rate (***μ***_**max**_) and lag phase (*λ*). The software Matlab R2010a (The MathWorks, USA) was used. For the analysis of* E. coli* reduction during the processing of salami was applied the model developed by Coroller et al. [21].

### 2.6. Statistical Analysis

This experiment was based on a completely randomized design with 3 replicates corresponding to 3 batches of salami for fatty acid analysis by GC-MS. For each TFA from each treatment at the evaluated and time points was compared through independent analysis by Duncan's test.

For the analysis of the concentration of myoglobin forms, 3 samples of 3 different batches of salami were analyzed for each treatment and processing time. Percentages of chemical forms of myoglobin were compared for each treatment using the F-test (P<0.05) and Duncan's test. The parameters obtained from the mass spectra and the analyses of the MDSC thermograms were compared for each treatment through the analysis of variance (P<0.05) and Duncan's test.

Microbial growth and* E. coli *reduction were evaluated for 2 independent samples from 3 different batches of salami for each treatment and processing time. The growth parameters were compared for each treatment through the analysis of variance (P<0.05) and Tukey's test. The statistical software SAS 9.2 (32) was used (SAS Institute Inc., Cary, NC, USA).

## 3. Results and Discussion

### 3.1. Changes in the Total Fatty Acid (TFA) Profile

The profiles of total fatty acids (TFA) during the processing of salami are expressed in mg/g of fat and are shown in Table 1. The concentration of the saturated fatty acids (SFA) myristic acid (C14:0) and palmitic acid (C16:0) during processing with the addition of the starter culture did not change significantly (P>0.05) as a function of the addition of NaNO_2_. The SFA myristic and stearic were higher abundance in the starter culture treatments; this finding was in concordance with results reported by Aksu and Kaya [22] on Pastirma (Turkish Dry Meat Product) where the SFA concentration was lower in control group than in treatments with starter culture composed by* Staphylococcus xylosus* +* Lactobacillus sakei* and* Staphylococcus carnosus* +* Lactobacillus pentosus*.

Regarding the concentration of monounsaturated fatty acids (MUFA), palmitoleic acid (C16:1) exhibited higher concentration in the postfermentation and postripening phases for treatment C^+^. The MUFA oleic acid (C18:1) had the highest concentrations in the control treatments and no differences (P>0.05) were associated with the addition of NaNO_2_. For the* Ls-Sc *and* Ls-Sc*^+^, there were no significant differences (P>0.05) in the concentration of the MUFA vaccenic acid (C18:1 trans-11) after the 7th day of ripening.

The polyunsaturated fatty acids (PUFA) linoleic acid and *γ*- linolenic acid were not identified in stages prior to prefermentation. During the ripening stage,* Ls-Sc*^+^ showed the highest concentration of this PUFA compared to treatment C, which could be correlated with the greater population density of the starter culture. According to Talon et al. [23]* S. carnosus *inhibited the oxidation of linoleic and linolenic acids in a pork fat but LAB such as* L. sakei, L. curvatus, *and* P. pentosaceus *did not present an antioxidant effect on linoleic acid* in vitro*. Zanardi et al. [24] did not find significant differences on total fatty acids profile for different fermented sausages with different concentrations for NaNO_2_. However, these authors reported differences on polyunsaturated fatty acids (PUFAs) on the meat product associated with starter culture strain composition, where a starter culture composed by* Lactobacillus* spp. and* S. carnosus *presents lower PUFAs concentration than meat products with starter cultures composed by* L. curvatus* and* Micrococcus varians*;* L. sakei, P. pentosaceus, S. xylosus*, and* M. varians*; and* L. sakei*,* P. pentosaceus*,* S. xylosus*,* S. carnosus*, and* M. varians*.

In contrast, the PUFA eicosadienoic acid was not found during the postripening stage; this result is concordant with the results reported by Qiu et al. [25] where decrease the proportion of the long chain PUFAs such as C20:4, C20:2, and C20:1 during cantonese sausage processing. This behavior is due to the oxidation of polyunsaturated fatty acids releasing aldehydes and ketones that affect the flavor of the final product during ripening [26].

During the post-fermentation stage, treatments with and without starter culture (addition of 50 ppm NaNO_2_) generated the highest concentration of SFA myristic acid and MUFA palmitoleic acid. These results could be related to the high *μ*_max_ values of CNS probably dominated by* S. carnosus* in treatments with the addition of NaNO_2_. The SFA/ (MUFA + PUFA) ratio was higher during the entire process for treatments with the starter culture compared to the controls; however, this behavior was not associated with the addition of NaNO_2_. The TFA ratio increased during the post-fermentation stage and decreased during ripening for the* Ls-Sc *and* Ls-Sc*^+^. The results previously reported by Marco et al. [27] are indicative that the total polyunsaturated fatty acid concentration change as a function of processing time on different lipid fraction (free fatty acids, phospholipids and triglycerides), where the free fatty acids within the total fatty acids present high increase on the concentration and proportion of PUFAs. Martín-Sánchez et al. [28] report PUFAs increase in dry-cured fermented Spanish sausage inoculated with* L. sakei *and* S. carnosus* as the starter culture during ripening, but during fermentation, there is a high oxidation rate of unsaturated triglycerides.

### 3.2. Modulated Differential Scanning Calorimetry (MDSC) of Salami Lipids between the Pre-Fermentation and Post-Ripening Stages for the Ls-Sc^+^

**Figure 1:**
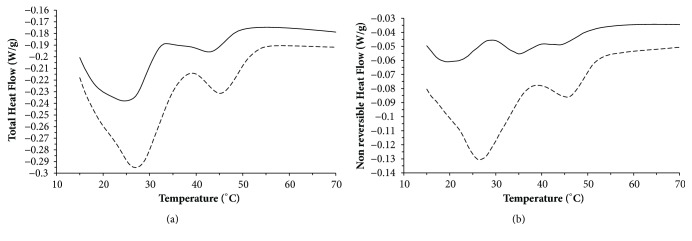
Modulated Differential Scanning Calorimetry (MDSC) curves indicating exothermic/endothermic total heat flow (a) and nonreversible heat flow (b) for salami lipid fraction in prefermentation stage (--) and postripening stage (- -).

The lipid thermograms of salami for the* Ls-Sc*^+^are shown in Figure 1. Transitions in the total heat flow (Figure 1(a)) show combination on reversing and nonreversing curves (Figure 1(b)), which indicate that the transitions show a combination of melting and crystallization of fatty acids between the prefermentation and postripening stages; and their contribution can be determined by enthalpy [29]. For this reason, a higher enthalpy for transitions in the nonreversible heat flow in the prefermentation stage could be related to crystallization and fatty acids fusion events as the main energetic contribution in this processing stage.

For the nonreversible and total heat flow, an increase was observed (P<0.05) on T_o_ and ΔH in the post-ripening stage compared with prefermentation stage (Table 2). The increase on enthalpy (ΔH) can be related to the increase in the concentration of primary and secondary products of the lipid oxidation and free fatty acids [14] during the salami processing. Furthermore, in the nonreversible period three transitions were observed for prefermentation stage and two transitions for the postripening stage (Table 2). This behavior indicates a kinetic process on salami lipids, and it can be associated with changes on the enthalpy relaxation reflecting structural reorganization and modifications [29].

Transitions with similar T_o_ had an increment on Cp in the postripening stage respect to the pre-fermentation stage; this was more evident for nonreversible heat flow (Table 3). The phenomena observed is comparable to the results reported by Samyn et al. [29]; Hidalgo and Zamora [30]; Coupland and McClements [31], where the increment on Cp is in function of the increase of the degree of unsaturation and the lipid autoxidation reactions developed during salami ripening. This is also seen in the results for the treatment* Ls-Sc*^+^ which are in concordance with the decreasing levels of SFA stearic acid, with significant differences (P<0.05) observed between the prefermentation and post-ripening periods. The concentration of PUFAs (linoleic and *γ*-linolenic acid) increased significantly (P<0.05) during the postripening period and the increment on lipid oxidation related to the relative abundance of MMb.

### 3.3. Changes in the Concentrations of Myoglobin Forms

The concentrations of DMB, OMB and MMB are shown in Table 4. According to Pérez-Alvarez* et al*. [32], the decrease in the concentration of OMB in the treatments with the addition of NaNO_2_ was inverse to the formation of nitrosomyoglobin complexes. For treatments C^+^,* Ls-Sc, *and C the decrease was more evident in the OMB related with the processing time. However, in the treatment with NaNO_2_ the OMB decrease was perceived from day 7 of processing (ripening), whereas for* Ls-Sc *and C this decrease was gradual from first day (fermentation). The oxidation of OMB increases proportionally at a lower pH, lower partial oxygen pressure, and lipid oxidation [8].

For all treatments, the concentration of MMB increased as a function of the processing time (Table 4). The highest concentration of MMB with significant differences (p<0.05) was for C^+^ and C. Consistent with Tang et al. [33], these changes in myoglobin oxidation were directly related to lipid oxidation and its products (i.e., aldehydes) that initiate conformational changes in myoglobin and cause an increase in the oxidation of the haem group and, therefore, it is darkening [18]. Moreover, the lipid oxidation during salami processing could be related with an increase on NaCl concentration; according to Ying* et al.* [34], who reported in dry-cured goose sausages that this reaction during the ripening stage could be due to the pro-oxidative effect of salt. On the other hand, the increase in NaCl concentration increases the activity of endogenous enzyme lipoxygenase (LOX) involved in lipid oxidation.

The OMB oxidation to MMB generates reactive intermediates capable of enhancing further oxidation of unsaturated fatty acids; in this sense, a superoxide anion is achieved which one dismutase to hydrogen peroxide. The latter can react with MMB concurrently generated in this oxidation sequence to form an activated MMB complex capable of enhancing lipid oxidation that is attributed to ferryl myoglobin [8]. This phenomenon indicates that the starter culture addition could generate an antioxidant effect, associated with the* S. carnosus *capacity to consume the free oxygen and nitrate reductase, catalase, and SOD activities and for* L. sakei *the nitrite reductase and catalase activities. However, the 50 ppm of NaNO_2 _addition did not accomplish a clear effect on the decrease of MMB formation. Furthermore, during the salami processing the treatments with the addition of a starter culture acquired higher concentration of PUFAs than the control treatments; this behavior is also related to an antioxidant capacity from the starter culture, because primary and secondary products derived from unsaturated fatty acids oxidation can enhance myoglobin oxidation [8].

### 3.4. Microbial Dynamics during Salami Processing

**Figure 2:**
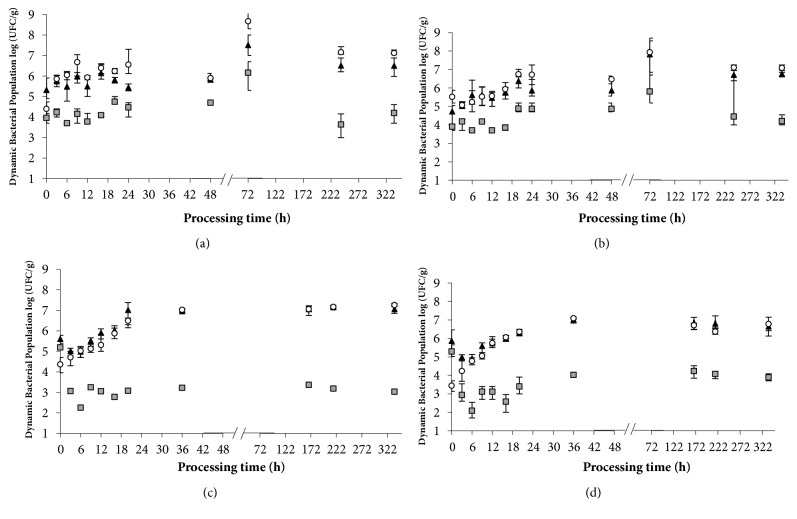
Dynamics of the microbial population during the processing of salami: fermentation at 21°C from 0 to 48 h and ripening at 17°C to 336 h (14 days). CNS (grey square), total bacterial count (black triangle), and LAB (white circle). Treatments* Ls-Sc* (a),* Ls-Sc*^+^ (b), C (c), and C^+^ (d). The bars indicate the confidence intervals.

The dynamics of microbial growth during the salami processes are shown in Figure 2. The population density of CNS was maintained for the treatment with the starter culture (Figures 2(a) and 2(b)), whereas for the control treatment a decrease in the microorganism population was observed during the first 6 hours of fermentation of the salami (Figures 2(c) and 2(d)). In accordance with previous reports by Hospital et al. [35], the addition of low levels of nitrite (<60ppm) can promote microbial growth under low levels of oxygen pressure (inner part of the meat product) also acting as a terminal acceptor of electrons, stimulating reductase activity. Microorganisms from the Staphylococcal genus can rapidly grow under aerobic conditions (surface), and their reductase activity decreases with high oxygen pressure; in this way, excess nitrite may cause the inhibition of growth associated with the interference of the electron transport chain. Furthermore, in the treatments* Ls-Sc *and* Ls-Sc*^+^, oxygen consumption (reduction of redox potential) and nitrate reductase activity is expected, according to Tjener et al. [36]. This phenomenon generates a positive influence on color formation and a negative influence on lipid oxidation.

In all treatments of this study, there were no differences in the bacterial concentrations of BAL and CNS with the addition of 50 ppm NaNO_2_ during the process of salami. The influence on changes in lipid fraction could be related to the fatty acid biosynthesis pathway by* S. carnosus* [37], where branched-chain fatty acids represent the majority of fatty acids produced by* S. carnosus*. However, for* L. sakei *were not found enzymes implicated with fatty acid biosynthesis or lipid transformation such as elongases or desaturases.

For the total LAB, there were no differences (P>0.05) in the *μ*_max_ between the* Ls-Sc, Ls-Sc*^+^, and C^+^ treatments; however, *μ*_max_ decreased to 0.003 h^−1^ following treatment C. In* Ls-Sc*^+^, *λ* increased to 14.7 h (Table 5). The population density of LAB in all treatments increased until 72 h of salami processing to approximately of 7 log CFU.g^−1^ and the control treatments was maintained until the post-ripening stage, while for* Ls-Sc *and* Ls-Sc*^+^ from 7th day (ripening) began to slight decrease, this behavior is concordant with results reported by Lorenzo et al. [38] where LAB population declines during ripening associated to the reduction of fermentable carbohydrates and a_w_. The growth rate (*μ*_max_) of aerobic mesophiles was determined as the total bacterial count. The C and C^+^ treatments showed a higher *μ*_max_; for these treatments, the dominant microbiota was native to the meat and adapted to the environment and therefore could present a higher growth rate. In contrast, no differences were associated with the addition of 50 ppm NaNO_2_ for the evaluated treatments or the cell concentrations in the postripening stage (P>0.05).

### 3.5. Validating the Reduction of E. coli during Salami Processing for Treatments with Starter Culture

**Figure 3:**
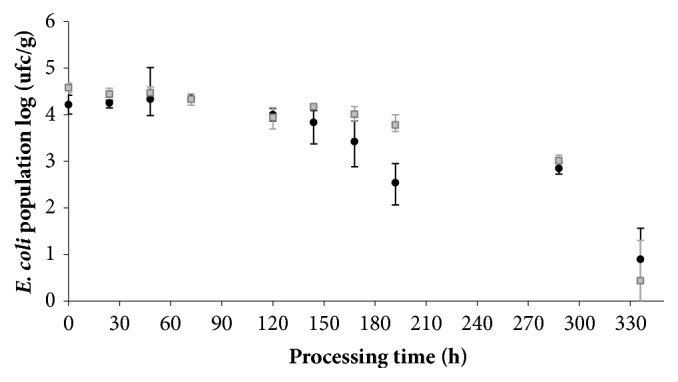
Dynamics of* E. coli *population during the processing of salami: fermentation at 21°C from 0 to 48 h and ripening at 17°C to 336 h (14 days). Treatments:* Ls-Sc* (grey square) and* Ls-Sc*^+^ (black circle). The bars indicate the confidence intervals.

The* E. coli *reduction during salami processing is shown in Figure 3.* E. coli *was reduced in the treatment* Ls-Sc *in 4.15±0.75 log CFU/g (90.5%) and in* Ls- Sc*^+^ in 4.22±0.81 log CFU/g (78.7%). The inactivation parameters showed that (*α*) was 15.99 for* Ls-Sc *and 19.47 for* Ls- Sc*^+^ implying greater resistance in the first phase of the salami processing for treatment with NaNO_2_. The parameter (*p) *was 6 for* Ls-Sc* and 1.79 for* Ls- Sc*^+^, thus* E. coli *treatment resistance related with adaptation speed was reduced in the NaNO_2_ addition. Inactivation rate (*δ*), for* Ls- Sc *(*δ*_1_) was 269.55 h^−1^ and (*δ*_2_) was 544.31 h^−1^, and for* Ls- Sc*^+^ (*δ*_1_) was 169.47 h^−1^ and (*δ*_2_) was 321.88 h^−1^. As a consequence, the NaNO_2_ addition reduces decimal inactivation time for both subpopulation of* E. coli*. Therefore, this behavior indicates the variability of the stress response of* E. coli *attributed to multiple factors combined as direct and indirect inhibition caused by the starter culture addition, NaNO_2_, pH, and a_w_. Holck et al. [39] mentioned that* E. coli *is generally inhibited in fermented sausages due to a combination of several hurdles: high salt, the presence of nitrite curing salt, low a_w_, decrease of redox potential, the growth of competitive starter culture, and a decrease in pH. Wang et al. [40] found the growth inhibition of* E. coli *during fermentation and ripening of Chinese fermented sausages by* L. sakei *and 100 ppm of NaNO_2_ addition. Results by Casey and Condon [41] reported that* E. coli* O157: H45 inhibition rate was faster with 50 ppm NaNO_2_ addition than without NaNO_2_ during processing of sausage fermented with* P. acidilactici.*

## 4. Conclusions

The addition of starter culture in salami processing increase the concentration of PUFAs (linoleic, *γ*- linolenic, and eicosadienoic), in contrast to the MMb concentration, which was lower. This latest concentration could be associated with an antioxidant effect produced by* S. carnosus* in the starter culture. On the other hand, the addition of 50 ppm NaNO_2_ did not have influence in the concentrations of the TFAs and MMb formation. Moreover, the starter culture without and with 50 ppm NaNO_2_ addition allowed the reduction of 4 log CFU/g of* E. coli*. However, the stress response of* E. coli* showed a broad variability because of bacteria inhibition (attributed to multiple factors combined). Therefore, these results allow analyzing changes during salami processing in a holistic form, where the application of starter culture with low nitrite concentrations could be a strategy for the meat industry to reduce this additive.

## Figures and Tables

**Table 1:** Changes in total fatty acids (TFAs) during salami processing expressed in mg/g of fat. Treatments with (*Ls- Sc*) and without (C) starter addition; the superscript ^**+**^ indicates the addition of 50 ppm NaNO_2_.

**TFA**	**Treatments**	**Processing time (Days)**											
		**0**		**2**		**5**		**7**		**10**		**14**	
Myristic acid (C14:0)	*Ls-Sc*	4.18 ± 0.30^a^	^*A*^	1.62 ± 0.19^c^	^*D*^	3.11 ± 0.21^a^	^*C*^	3.71 ± 0.30^a^	^*B*^			3.45 ± 0.31^a^	^*B,C*^
	*Ls-Sc* ^+^	3.68 ± 0.14^a^	^*A,B*^	3.68 ± 0.34^b^	^*A,B*^	3.20 ± 0.00^a^	^*B*^	3.36 ± 0.16^a,b^	^*B*^			4.24 ± 0.81^a^	^*A*^
	C	3.63 ± 0.14^a^	^*A,B*^	3.23 ± 0.48^b^	^*B,C*^			2.41 ± 0.72^b,c^	^*C*^	0.00 ± 0.00^b^	^*D*^	4.09 ± 0.37^a^	^*A*^
	C^+^	3.60 ± 0.86^a^	^*C*^	5.54 ± 0.00^a^	^*A*^			2.36 ± 0.59^c^	^*D*^	3.38 ± 0.69^a^	^*C*^	4.46 ± 0.19^a^	^*B*^

Palmitoleic acid (C16:1)	*Ls-Sc*	3.72 ± 0.24^b,c^	^*A*^	1.27 ± 0.06^c^	^*D*^	3.09 ± 0.06^a^	^*B*^	2.56 ± 0.34^b^	^*C*^			2.75 ± 0.24^b^	^*B*^
	*Ls-Sc* ^+^	3.57 ± 0.11^c^	^*A*^	2.73 ± 0.60^b^	^*B,C*^	2.03 ± 0.71^b^	^*D*^	3.31 ± 0.11^a^	^*A,B*^			2.48 ± 0.49^b^	^*C,D*^
	C	4.82 ± 0.48^a^	^*A*^	2.31 ± 0.39^b^	^*B*^			0.00 ± 0.00^d^	^*C*^	0.00 ± 0.00^b^		2.71 ± 0.65^b^	^*B*^
	C^+^	4.35 ± 0.40^a,b^	^*B,C*^	4.59 ± 0.00^a^	^*A,B*^			1.26 ± 0.00^c^	^*D*^	4.99 ± 0.96^a^	^*A*^	3.94 ± 0.35^a^	^*C*^

Palmitic acid (C16:0)	*Ls-Sc*	56.15 ± 1.10^b^	^*A*^	56.33 ± 2.85^c^	^*A*^	54.56 ± 3.36^b^	^*A*^	57.25 ± 4.95^b^	^*A*^			53.48 ± 3.60^b^	^*A*^
	*Ls-Sc* ^+^	58.60 ± 1.26^a,b^	^*B*^	57.65 ± 4.25^c^	^*B*^	63.77 ± 4.37^a^	^*A*^	50.17 ± 1.49^c^	^*C*^			56.05 ± 2.54^b^	^*B*^
	C	57.19 ± 2.94^a,b^	^*C*^	72.76 ± 6.74^b^	^*A,B*^			68.94 ± 3.97^a^	^*B*^	80.59 ± 2.68^a^	^*A*^	71.60 ± 5.82^a^	^*B*^
	C^+^	59.81 ± 2.09^a^	^*A*^	87.72 ± 1.48^a^	^*B*^			61.20 ± 0.10^d^	^*C*^	64.54 ± 3.00^b^	^*D*^	72.56 ± 2.81^a^	^*E*^

Oleic acid (C18:1)	*Ls-Sc*	104.68 ± 0.92^a^	^*A*^	91.62 ± 8.03^c^	^*C*^	96.79 ± 1.46^a^	^*B*^	92.63 ± 6.54^a^	^*B.C*^			91.10 ± 6.00^b^	^*C*^
	*Ls-Sc* ^+^	107.12 ± 3.28^a^	^*A*^	85.17 ± 8.31^c^	^*B*^	86.94 ± 8.17^b^	^*B*^	91.17 ± 4.75^a^	^*B*^			90.24 ± 3.66^b^	^*B*^
	C	104.49 ± 4.21^a^	^*C*^	120.2 ± 7.87^b^	^*A,B*^			123.25 ± 3.70^b^	^*D*^	121.7 ± 7.74^a^	^*A*^	110.28 ± 9.05^a^	^*B,C*^
	C^+^	105.28 ± 4.33^a^	^*C*^	125.8 ± 1.40^a^	^*A*^			116.88 ± 0.63^b^	^*D*^	114.9 ± 3.37^a^	^*B*^	108.34 ± 4.99^a^	^*C*^

Vaccenic acid (C18:1 *trans*-11)	*Ls-Sc*	4.41 ± 0.68^b^	^*A*^	1.68 ± 0.18^b^	^*C*^	1.42 ± 0.00^b^	^*C*^	3.09 ± 0.45^b^	^*B*^			3.14 ± 0.30^a^	^*B*^
	*Ls-Sc* ^+^	4.38 ± 0.23^b^	^*A*^	2.76 ± 0.00^a^	^*B,C*^	3.48 ± 1.25^a^	^*A,B*^	3.50 ± 0.03^b^	^*A,B*^			2.43 ± 0.71^a^	^*C*^
	C	5.68 ± 0.88^a^	^*B*^	2.71 ± 0.13^a^	^*B,C*^			1.16 ± 0.61^a^	^*A*^	4.45 ± 0.10^b^	^*B,C*^	1.40 ± 0.02^b^	^*B,C*^
	C^+^	4.42 ± 0.08^b^	^*C*^	0.00 ± 0.00^c^	^*C*^			1.55 ± 0.83^a^	^*A*^	2.51 ± 0.41^a^	^*B*^	1.37 ± 0.00^b^	^*C*^

Stearic acid (C18:0)	*Ls-Sc*	22.40 ± 1.38^a^	^*A*^	12.60 ± 2.18^a^	^*C*^	14.59 ± 1.41^a^	^*B*^	14.83 ± 0.26^a^	^*B*^			16.18 ± 0.33^a^	^*B*^
	*Ls-Sc* ^+^	18.62 ± 5.60^a,b^	^*A*^	11.12 ± 1.80^a^	^*C*^	9.39 ± 0.09^b^	^*C*^	13.79 ± 0.81^a^	^*B*^			14.16 ± 0.05^c^	^*B*^
	C	19.63 ± 0.72^b^	^*A*^	12.48 ± 0.55^a^	^*B*^			10.04 ± 0.62^b^	^*C*^	7.21 ± 1.87^b^	^*D*^	15.07 ± 0.16^b^	^*E*^
	C^+^	19.74 ± 0.28^b^	^*A*^	12.62 ± 1.10^a^	^*C*^			11.59 ± 0.51^b^	^*D*^	10.02 ± 0.81^a^	^*A*^	15.75 ± 0.43^a^	^*B*^

Linoleic acid (C18:2)	*Ls-Sc*	0.00 ± 0.00		2.07 ± 0.32^b^	^*B*^	4.19 ± 0.65^b^	^*A*^	1.05 ± 0.06^c^	^*C*^			1.62 ± 0.22^c^	^*B*^
	*Ls-Sc* ^+^	0.00 ± 0.00		2.03 ± 0.85^b^	^*C*^	7.75 ± 2.94^a^	^*A,B*^	9.88 ± 2.47^b^	^*A*^			6.65 ± 0.17^a^	^*B*^
	C	0.00 ± 0.00		4.04 ± 0.24^a^	^*B*^			1.74 ± 0.60^a^	^*A*^	0.00 ± 0.00		0.00 ± 0.00^d^	^*C*^
	C^+^	0.00 ± 0.00		4.25 ± 0.98^a^	^*A*^			2.22 ± 0.79^a^	^*B*^	0.00 ± 0.00		2.58 ± 0.00^b^	^*B*^

*γ*-linolenic acid (C18:3)	*Ls-Sc*	0.00 ± 0.00		0.00 ± 0.00		1.49 ± 0.30^b^	^*C*^	3.17 ± 0.02^b^	^*B*^			26.62 ± 1.29^a^	^*A*^
	*Ls-Sc* ^+^	0.00 ± 0.00		0.00 ± 0.00		14.16 ± 0.50^a^	^*B*^	14.30 ± 0.37^a^	^*C*^			30.32 ± 6.66^a^	^*A*^
	C	0.00 ± 0.00		0.00 ± 0.00				1.62 ± 0.31^c^	^*B*^	3.91 ± 0.21^a^	^*A*^	1.37 ± 0.18^b^	^*C*^
	C^+^	0.00 ± 0.00		0.00 ± 0.00				0.00 ± 0.00^d^	^*B*^	0.00 ± 0.00^b^	^*B*^	4.94 ± 0.00^b^	^*A*^

Eicosadienoic acid (C20:2)	*Ls-Sc*	1.20 ± 0.00^c^	^*C*^	48.72 ± 2.74^a^	^*A*^	20.91 ± 4.18^b^	^*B*^	21.48 ± 3.81^b^	^*B*^			0.00 ± 0.00^b^	^*C*^
	*Ls-Sc* ^+^	1.42 ± 0.00^b^	^*C*^	39.21 ± 1.67^b^	^*A*^	40.96 ± 4.85^a^	^*A*^	27.80 ± 3.69^a^	^*B*^			0.00 ± 0.00^b^	^*C*^
	C	0.00 ± 0.00^d^	^*C*^	0.00 ± 0.00^c^	^*C*^			3.16 ± 0.56^c^	^*A*^	0.00 ± 0.00^b^	^*C*^	1.57 ± 0.44^a^	^*B*^
	C^+^	2.56 ± 0.00^a^	^*B*^	0.00 ± 0.00^c^	^*D*^			3.78 ± 0.19^c^	^*A*^	1.25 ± 0.00^a^	^*C*^	0.00 ± 0.00^b^	^*D*^

Values are means ± SEM of three replicates.

^a-d^ Different letters between rows (treatments) for each TFA show significant differences P<0.05 by Duncan.

^A-D^ Different letters between columns (processing time) for each TFA show significant differences P<0.05 by Duncan.

**Table 2:** Onset temperature of the endothermic peak (T_o_), maximum temperature (T_max_), and enthalpy (ΔH) of the lipid fractions of salami treatment *Ls-Sc*^*+*^ in the pre-fermentation and postripening stages.

**Stage**	**Non-reversible heat flow**			**Total heat flow **		
	**T**_**o**_ ** (**°**C)**	**T**_**max**_ ** (**°**C)**	Δ**H (J/g)**	**T**_**o**_ ** (**°**C)**	**T**_**max**_ ** (**°**C)**	Δ**H (J/g)**
Pre-fermentation	15.750	22.380	1.275	16.750	24.820	4.884
	32.700	35.780	0.371	37.980	43.290	0.881
	80.140	84.280	0.031			

Post - ripening	16.187	25.290	5.495	19.195	27.650	6.610
	40.105	45.080	1.230	39.805	45.330	2.211

**Table 3:** Transitions of nonreversible and total heat flow, onset temperature of the endothermic peak (T_o_), midpoint temperature (T_m_), and specific heat (ΔCp) of lipid fractions of salami of treatment *Ls-Sc*^*+*^ in the prefermentation and postripening stages.

**Stage**	**Non-reversible heat flow**			**Total heat flow**		
	**T**_**o**_ ** (**°**C)**	**T**_**m**_ ** (**°**C)**	Δ**Cp (J/g **°**C)**	**T**_**o**_ ** (**°**C)**	**T**_**m**_ ** (**°**C)**	Δ**Cp (J/g **°**C)**
Pre-fermentation	13.140	13.260	0.076	16.220	16.740	0.443
	16.430	16.860	0.098	27.510	28.670	0.538
	23.470	25.120	0.074	39.080	40.700	0.054
	28.637	29.033	0.020	44.110	46.450	0.227
	31.263	31.685	0.020	64.070	75.930	0.054
	43.695	47.340	0.166	81.410	93.180	0.096
	79.490	82.610	0.011			
	93.840	96.820	0.024			

Post-ripening	13.270	13.400	0.269	16.105	16.775	0.642
	17.380	16.650	0.134	29.230	31.150	0.759
	23.780	25.383	0.134	40.160	42.385	0.208
	27.445	29.130	0.305	47.105	48.905	0.360
	40.670	42.480	0.102	62.580	64.930	0.020
	47.075	49.535	0.262	82.845	92.950	0.046
	61.138	63.455	0.031			
	94.485	97.095	0.023			

**Table 4:** Myoglobin species (%), deoxymyoglobin (DMB), oxymyoglobin, (OMB) and metmyoglobin (MMB). Treatments with (*Ls- Sc*) and without starter culture addition (C); the superscript ^+^ indicates the addition of 50 ppm NaNO_2_.

**Myoglobin Form (%)**	**Processing Time (Days)**	**Treatment**											
		***Ls-Sc*** ^***+***^			***Ls-Sc***			**C** ^**+**^			**C**		
**DMB**	0	32.06 ± 0.42	^a^	^A^	28.86 ± 0.84	^c^	^B^	30.18 ± 0.65	^a^	^C^	27.45 ± 0.65	^c^	^D^
	1	29.18 ± 0.26	^d^	^A^	29.04 ± 0.15	^c^	^A^	26.04 ± 0.10	^b^	^C^	27.54 ± 0.05	^c^	^B^
	2	29.20 ± 0.52	^d^	^A^	28.42 ± 0.20	^c^	^B^	26.95 ± 0.99	^c^	^C^	32.26 ± 0.50	^a^	^D^
	3	29.84 ± 0.24	^c^	^A^	30.80 ± 0.42	^b^	^B^	24.14 ± 0.42	^d^	^C^	27.89 ± 0.62	^c^	^D^
	7	31.55 ± 0.12	^b^	^A^	30.66 ± 0.75	^b^	^B^	31.23 ± 0.70	^e^	^A,B^	28.67 ± 0.68	^b^	^C^
	14	28.75 ± 0.22	^e^	^A^	33.20 ± 0.93	^a^	^B^	28.61 ± 0.37	^a^	^A^	27.09 ± 0.15	^c^	^C^

**OMB**	0	18.56 ± 0.56	^d^	^A^	25.43 ± 0.93	^a^	^B^	20.58 ± 0.09	^a^	^C^	22.47 ± 0.84	^a^	^D^
	1	20.80 ± 0.87	^b^	^A^	25.75 ± 0.43	^a^	^B^	24.21 ± 0.40	^b^	^C^	21.97 ± 0.48	^a^	^D^
	2	21.75 ± 0.69	^a^	^A^	22.60 ± 0.75	^b^	^B^	25.78 ± 0.86	^c^	^C^	17.80 ± 0.08	^c^	^D^
	3	21.76 ± 0.55	^a^	^A^	18.92 ± 0.24	^d^	^B^	27.72 ± 0.61	^d^	^C^	22.39 ± 0.67	^a^	^D^
	7	17.94 ± 0.46	^e^	^B^	19.94 ± 0.94	^c^	^A^	16.60 ± 0.75	^e^	^C^	19.84 ± 0.19	^b^	^A^
	14	20.13 ± 0.51	^c^	^A^	17.10 ± 0.79	^e^	^B^	16.01 ± 0.92	^e^	^C^	17.05 ± 0.67	^d^	^C^

**MMB**	0	49.98 ± 0.90	^b^	^A^	45.37 ± 0.59	^e^	^B^	49.66 ± 0.19	^b^	^A^	50.01 ± 0.68	^c^	^A^
	1	50.34 ± 0.69	^b^	^A^	45.17 ± 0.08	^e^	^C^	49.57 ± 0.43	^b^	^B^	50.70 ± 0.48	^b^	^A^
	2	49.17 ± 0.21	^c^	^B^	48.99 ± 0.23	^d^	^B^	46.98 ± 0.50	^d^	^C^	49.93 ± 0.72	^c^	^A^
	3	48.60 ± 0.71	^d^	^A^	50.67 ± 0.73	^a^	^B^	47.65 ± 0.01	^c^	^C^	49.58 ± 0.64	^c^	^D^
	7	51.00 ± 0.86	^a^	^A^	49.52 ± 0.55	^c^	^B^	52.69 ± 0.39	^a^	^C^	51.76 ± 0.69	^a^	^D^
	14	51.09 ± 0.40	^a^	^A^	50.14 ± 0.05	^b^	^B^	52.32 ± 0.65	^a^	^C^	51.70 ± 0.08	^a^	^D^

Values are means ± SEM of nine samples with five replicates.

^a-e^ Different letters between rows (processing time) for each Myoglobin species show significant differences P<0.05 by Duncan.

^A-D^ Different letters between columns (treatment) for each processing time and Myoglobin species show significant differences P<0.05 by Duncan.

**Table 5:** Biokinetic parameter estimation using Baranyi and Roberts's model.

**Microorganism**	**Treatment**	***μ***_**max**_ ** (h** ^**-1**^ **)**		***λ*** **(h)**
CNS	*Ls-Sc*	-0.0006 ± 0.0000	^g^	- *∗*
	*Ls-Sc* ^+^	0.037 ± 0.002	^e,f,g^	- *∗*
	C	0.0007 ± 0.000	^g^	- *∗*
	C^+^	0.164 ± 0.035	^a^	- *∗*

LAB	*Ls-Sc*	0.029 ± 0.001	^e,f,g^	- *∗*
	*Ls-Sc* ^+^	0.067 ± 0.001	^c,d,e^	14.691 ± 0.200
	C	0.003 ± 0.0004	^g^	- *∗*
	C^+^	0.052 ± 0.003	^e,f^	- *∗*

Total count	*Ls-Sc*	0.019 ± 0.003	^f,g^	- *∗*
	*Ls-Sc* ^+^	0.034 ± 0.003	^e,f,g^	- *∗*
	C	0.108 ± 0.007	^b,c^	- *∗*
	C^+^	0.098 ± 0.012	^c,d^	- *∗*

Values are means ± SEM with six replicates.

Different letters show significant differences P<0.05 by Tukey test.

-*∗* Parameter not calculated (*λ*< 3h).

## Data Availability

The data used to support the findings of this study are available from the corresponding author upon request.
